# Machine Learning to Predict the Response to Lenvatinib Combined with Transarterial Chemoembolization for Unresectable Hepatocellular Carcinoma

**DOI:** 10.3390/cancers15030625

**Published:** 2023-01-19

**Authors:** Jun Ma, Zhiyuan Bo, Zhengxiao Zhao, Jinhuan Yang, Yan Yang, Haoqi Li, Yi Yang, Jingxian Wang, Qing Su, Juejin Wang, Kaiyu Chen, Zhengping Yu, Yi Wang, Gang Chen

**Affiliations:** 1 Department of Epidemiology and Biostatistics, School of Public Health and Management, Wenzhou Medical University, Wenzhou 325035, China; 2 Department of Hepatobiliary Surgery, The First Affiliated Hospital of Wenzhou Medical University, Wenzhou 325035, China; 3 Department of Oncology, The First Affiliated Hospital of Zhejiang Chinese Medical University, Hangzhou 310053, China; 4 Key Laboratory of Diagnosis and Treatment of Severe Hepato-Pancreatic Diseases of Zhejiang Province, The First Affiliated Hospital of Wenzhou Medical University, Wenzhou 325035, China

**Keywords:** machine learning, lenvatinib, transarterial chemoembolization, hepatocellular carcinoma, Shapley Additive exPlanation, treatment response

## Abstract

**Simple Summary:**

The objective response rate of lenvatinib combined with transarterial chemoembolization for unresectable hepatocellular carcinoma is unsatisfactory. We aimed to develop predictive models using demographic characteristics, pre-treatment serum biomarkers and tumor characteristics of unresectable hepatocellular carcinoma patients by five machine learning algorithms to predict the response under combined treatments. We identified the 10 most important predictors, including K, low-density lipoprotein, D-dimer, red blood cell, alanine aminotransferase, albumin, monocyte, tumor size, triglyceride, and age. In addition, we applied the Shapley Additive exPlanation to explain the best-performing random forest predictive model to provide a reasonable explanation of the efficacy prediction at an individualized level. The combination of machine learning and Shapley Additive exPlanation can provide valuable suggestions for clinical decision making.

**Abstract:**

Background: Lenvatinib and transarterial chemoembolization (TACE) are first-line treatments for unresectable hepatocellular carcinoma (HCC), but the objective response rate (ORR) is not satisfactory. We aimed to predict the response to lenvatinib combined with TACE before treatment for unresectable HCC using machine learning (ML) algorithms based on clinical data. Methods: Patients with unresectable HCC receiving the combination therapy of lenvatinib combined with TACE from two medical centers were retrospectively collected from January 2020 to December 2021. The response to the combination therapy was evaluated over the following 4–12 weeks. Five types of ML algorithms were applied to develop the predictive models, including classification and regression tree (CART), adaptive boosting (AdaBoost), extreme gradient boosting (XGBoost), random forest (RF), and support vector machine (SVM). The performance of the models was assessed by the receiver operating characteristic (ROC) curve and area under the receiver operating characteristic curve (AUC). The Shapley Additive exPlanation (SHAP) method was applied to explain the model. Results: A total of 125 unresectable HCC patients were included in the analysis after the inclusion and exclusion criteria, among which 42 (33.6%) patients showed progression disease (PD), 49 (39.2%) showed stable disease (SD), and 34 (27.2%) achieved partial response (PR). The nonresponse group (PD + SD) included 91 patients, while the response group (PR) included 34 patients. The top 40 most important features from all 64 clinical features were selected using the recursive feature elimination (RFE) algorithm to develop the predictive models. The predictive power was satisfactory, with AUCs of 0.74 to 0.91. The SVM model and RF model showed the highest accuracy (86.5%), and the RF model showed the largest AUC (0.91, 95% confidence interval (CI): 0.61–0.95). The SHAP summary plot and decision plot illustrated the impact of the top 40 features on the efficacy of the combination therapy, and the SHAP force plot successfully predicted the efficacy at the individualized level. Conclusions: A new predictive model based on clinical data was developed using ML algorithms, which showed favorable performance in predicting the response to lenvatinib combined with TACE for unresectable HCC. Combining ML with SHAP could provide an explicit explanation of the efficacy prediction.

## 1. Introduction

Hepatocellular carcinoma (HCC) is one of the most fatal malignancies and the predominant type of primary liver cancer (PLC), accounting for approximately 90% of PLC cases [1,2]. Currently, the incidence and disease burden of HCC are increasing annually, with roughly 850,000 new cases and 810,000 deaths occurring per year [3]. HCC patients have no significant symptoms at the early stage and are generally diagnosed at an advanced stage, leading to a loss of curative surgical treatment [4]. Patients with unresectable HCC have poor prognosis with limited effective treatment options [5]. Due to the limited treatment options for unresectable HCC patients, systemic therapies such as molecular target agents have become the primary treatment choices [5,6].

As a small molecule, multitarget, multireceptor tyrosine kinase inhibitor (TKI), lenvatinib has become a first-line therapy for HCC [7,8]. The previous randomized phase 3 trial by Masatoshi et al. confirmed that lenvatinib showed a higher objective response rate (ORR) and longer median overall survival (OS) and median progression-free survival (PFS) compared with sorafenib [9]. Similarly, transarterial chemoembolization (TACE) is another first-line treatment for intermediate stage and advanced HCC as well [10]. TACE could trigger tumor ischemic necrosis by infusing cytotoxic chemotherapeutic agents and delivering embolic components to the tumor artery [11]. However, TACE could potentially expose the tumor to a hypoxic state and induce the upregulation of vascular endothelial growth factor (VEGF) and fibroblast growth factor (FGF), resulting in tumor revascularization [10]. Lenvatinib appears to be capable of inhibiting the revascularization caused by TACE, which targets VEGFR1-3 and FGFR1-4 [12,13]. Lenvatinib combined with TACE has shown a superior efficacy to sorafenib combined with TACE treatment, with a similar treatment safety [14]. However, the heterogeneity and chemoresistance of HCC prevent patients from significantly responding to lenvatinib combined with TACE, leading to an unsatisfactory ORR. Therefore, it is particularly critical to define the accurate predictive factors for the response to lenvatinib combined with TACE prior to treatment.

As an integral part of artificial intelligence (AI), machine learning (ML) can identify trends and patterns by inputting and processing data, setting up different algorithms, and applying computer analysis [15]. ML provides significant advantages over traditional statistical methods for the analysis and evaluation of medical data and big data [16]. ML applications can encompass multiple fields in medical research, including distinguishing benign and malignant tumors, identifying potential biomarkers, screening patients, and disease prediction and diagnosis [17]. In particular, the prediction and diagnosis of diseases can be improved significantly by establishing and developing models with ML algorithms [18]. Since human diseases are commonly accompanied by complicated dynamic changes during progression that are virtually impossible to identify artificially, ML methods have been widely used in the diagnosis, treatment, and prognosis of various diseases [19].

There have been rare studies focusing on predicting the response of patients with unresectable HCC to lenvatinib combined with TACE using ML algorithms. Therefore, this study aimed to predict the efficacy of combination therapy for patients with unresectable HCC using ML algorithms based on clinical data.

## 2. Materials and Methods

### 2.1. Study Population

This study was conducted based on a real-world cohort of consecutive patients treated with lenvatinib combined with TACE from two medical centers (the First Affiliated Hospital of Wenzhou Medical University and the First Affiliated Hospital of Zhejiang Chinese Medical University). A total of 453 patients with unresectable HCC were retrospectively registered from January 2020 to December 2021. HCC was diagnosed radiologically or histologically following the Asian Pacific Association for the Study of the Liver (APSAL) [20]. The inclusion criteria were as follows: (1) HCC diagnosed by radiology or histology; (2) receiving lenvatinib combined with TACE treatment; (3) age > 18 years; (4) Eastern Cooperative Oncology Group performance status (ECOG-PS) grade 0–1; (5) Child–Pugh score ≤ 7. The exclusion criteria were as follows: (1) with other malignancies; (2) receiving other antitumor treatments; (3) unable to estimate lenvatinib combined with TACE treatment efficacy; (4) lost to follow-up; (5) incomplete clinical data. To avoid the bias between the data from the two medical centers, we performed a principal component analysis (PCA) on all 64 standardized clinical features to explore the bias. The study was conducted according to the ethical principles of the Declaration of Helsinki and was approved by the Ethics Committee of the local institutional review boards (KY2022-R095). Written informed consent was obtained from each patient at the first time of admission to hospital.

### 2.2. Treatment Protocol and Response Evaluation

Lenvatinib was orally administered within 7 days before or after the TACE therapy [21]. According to the guidelines, the dose of lenvatinib was 12 mg/d for patients weighing ≥60 kg or 8 mg/d for patients weighing <60 kg [8]. Lenvatinib combined with TACE therapy was discontinued when unacceptable adverse events or significant disease progression were observed, or the patient withdrew consent. The tumor response was assessed by experienced hepatobiliary surgeons C. G. (15 years of clinical experience) and Y. Z. P. (35 years of clinical experience) with radiological methods with reference to the modified RECIST (mRECIST) criteria within 4–12 weeks after combination therapy [22]. The tumor response included the following 4 categories: (1) complete response (CR)—disappearance of intratumoral arterial enhancement in typical target lesions and disappearance of atypical intrahepatic and extrahepatic target; (2) partial response (PR)—the sum of the diameters of the target lesions (including viable tumor diameters for typical intrahepatic target lesions and short axis diameters for nodal lesions) was reduced by at least 30%, referenced to the sum of the longest diameters at baseline; (3) progressive disease (PD)—the sum of the diameters of the target lesions (including viable tumor diameters for typical intrahepatic target lesions and short axis diameters for nodal lesions) was increased by at least 20% AND at least 5 mm, referenced to the nadir sum of the diameters at baseline; (4) stable disease (SD)—neither sufficient reduction to achieve PR nor sufficient increase to advance to PD. The objective response rate (ORR) and disease control rate (DCR) were significant indicators showing the antitumor effect of the combination therapy. ORR was defined as the proportion of patients showing CR and PR. DCR was defined as the proportion of patients showing CR, PR, and SD.

### 2.3. Data Acquisition, Preprocessing, and Feature Extraction

We combined the patients showing PD and SD into a nonresponse group (*n* = 91), and the patients achieving PR were listed as a response group (*n* = 34). We collected 64 clinical features in total, including demographic characteristics (age, gender, body mass index (BMI), smoking history, drinking history, hepatitis B virus (HBV) infection, hepatitis C virus (HCV), hypertensive history, diabetes history, heart disease history, nonalcohol fatty liver disease (NAFLD) history, and cirrhosis history), pretreatment serum biomarkers, and tumor characteristics from patients with unresectable HCC. We standardized the continuous variables to eliminate the influence of the distance between the sample data. The standardized data were calculated by subtracting the mean from the original data and dividing by the standard deviation. A random forest (RF) model was developed based on all 64 clinical features and selected top 40 features during the iterative process by recursive feature elimination (RFE). RFE is a wrapped feature selection algorithm, essentially a greedy algorithm intended to select the feature subset with the best performance. In the end, we developed the predictive model with the selected 40 features.

### 2.4. Machine Learning Approach

We aimed to predict whether patients with unresectable HCC would have a response to lenvatinib combined with TACE using ML algorithms. The predicted outcomes were binary classes: nonresponse and response. The clinical data were randomly split, with 70% as the training set and 30% as the testing set. The training set was used for data fitting, hyperparameter determination, and prediction models development, while the testing set was used to evaluate the performance of the prediction models. We applied the synthetic minority oversampling technique (SMOTE) algorithm for the sampling to overcome the imbalance of the samples in the training set [23]. We utilized the following five supervised ML algorithms to develop the predictive models: classification and regression tree (CART), adaptive boosting (AdaBoost), extreme gradient boosting (XGBoost), RF, and support vector machine (SVM). The hyperparameters were selected in each ML model by three repetitions of 10-fold cross-validation, and a grid search was applied to determine the hyperparameters yielding the best model performance. We evaluated the predictive performance of each ML model by plotting and comparing the area under the receiver operating characteristic curve (AUC) and calculated the accuracy, precision, recall, F1-score, sensitivity, specificity, positive predictive value (PPV), negative predictive value (NPV), and Matthews correlation coefficient (MCC) from the confusion matrix of the testing set. In addition, we performed unsupervised clustering of the included 125 patients with unresectable HCC by the K-means algorithm and visualized the clustering results after the dimensionality reduction using t-distributed stochastic neighbor embedding (t-SNE). To make the black-box effects of the ML models explainable, we further used Shapley Additive exPlanation (SHAP) values based on cooperative game theory to elaborate the global model structure by multiple local explanations [24]. All ML analyses were performed using Python (version 3.10.5) in the Microsoft VS Code (version 1.69.0) development environment, with Scikit-learn (version 1.1.1), xgboost (version 1.6.1), NumPy (version 1.22.4), pandas (version 1.4.3), matplotlib (version 3.5.2), and shap (version 0.41.0) modules, etc.

### 2.5. Statistical Analysis

The continuous variables were described as the mean ± standard deviation (SD) or median (interquartile range, (IQR)) based on the data distribution, and the Student’s *t*-test or Mann–Whitney U test were further applied to compare the differences between the training and testing sets. The categorical variables were described as the frequencies (percentages) and analyzed using the chi-square test or Fisher exact test. *p*-Value < 0.05 was considered statistically significant. All statistical analyses were conducted using the R program (R Foundation for Statistical Computing, version 4.2.1).

## 3. Results

### 3.1. Patient Characteristics and Treatment Response

**Figure 1:**
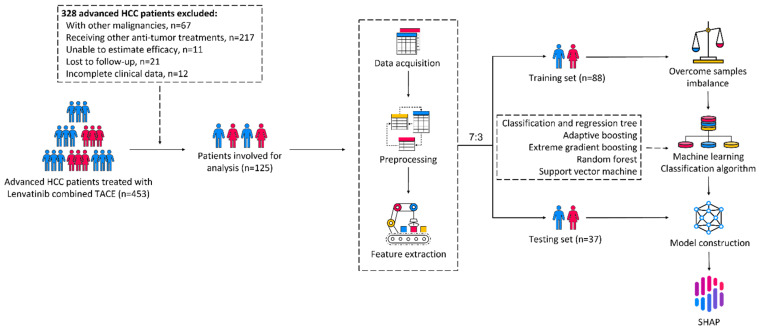
Flow chart of the study.

A flow chart of the study was shown in Figure 1.

We retrospectively collected data from 125 patients with unresectable HCC from January 2020 to December 2021. Among the 125 patients treated with lenvatinib combined with TACE, 42 (33.6%) patients showed PD, 49 (39.2%) showed SD, and 34 (27.2%) achieved PR. The ORR and DCR were 27.2% and 66.4%, respectively. We randomly divided the patients into a training set (*n* = 88) and testing set (*n* = 37) according to a ratio of 7:3 and compared all the features between the two groups. The demographic characteristics, pretreatment serum biomarkers, and tumor characteristics are shown in Table 1. The detailed raw data of 125 unresectable HCC patients were available in the Supplementary File S1.

The median age for the training set and testing set was 58.0 (IQR, 46.3–69.0) and 59.0 (IQR, 47.5–67.5) years, respectively. The red blood cell (RBC, *p* = 0.048), activated partial thromboplastin time (APTT, *p* = 0.040), direct bilirubin (D-BIL, *p* = 0.029), globulin (GLOB, *p* = 0.045), albumin/globulin (A/G, *p* = 0.011) showed statistical significance between the training and testing sets. Other features did not show statistical significance. Detailed information for the nonresponse group (PD + SD) and response group (PR) was listed in the Supplementary File S2 (Table S1). The PCA showed no significant bias between the data from the two medical centers (R = 0.0060, *p* = 0.461) in Supplementary File S2 (Figure S1).

### 3.2. Predictive Performance of the Machine Learning

We developed models based on the extracted 40 features with five types of ML classification algorithms, aiming to predict the efficacy of lenvatinib combined with TACE in unresectable HCC. The predictive performance matrix based on the testing set of the five models are shown in Table 2. The best hyperparameters and confusion matrix for five machine learning algorithms were listed in the Supplementary File S2 (Table S2) and Supplementary File S2 (Table S3), respectively.

**Figure 2:**
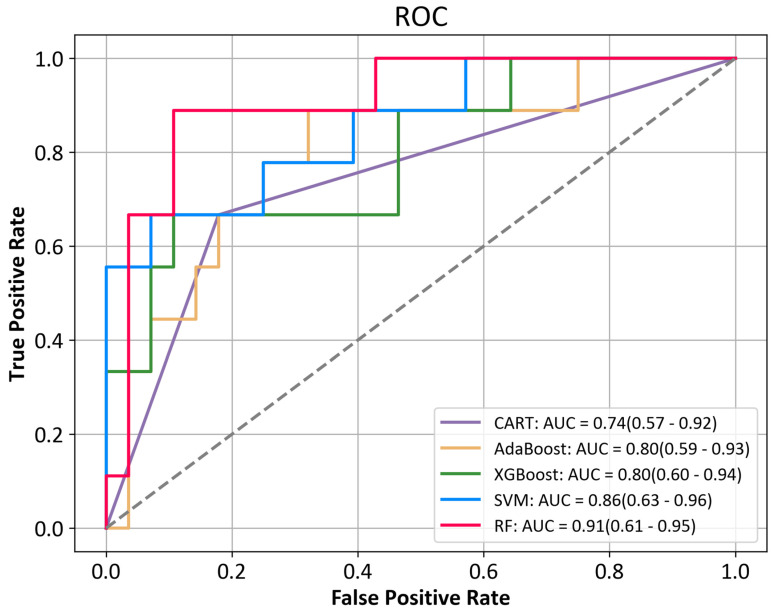
ROC curves for differentiating nonresponse and response using the machine learning algorithms. ROC, receiver operating characteristic; CART, classification and regression tree; AdaBoost, adaptive boosting; XGBoost, extreme gradient boosting; SVM, support vector machine; RF, random forest.

All of the ML models showed a moderate-to-excellent predictive performance (the AUCs ranged from 0.74 to 0.91). The SVM model and RF model showed the highest prediction accuracy (86.5%). All of the models were excellent in predicting the nonresponders to combined therapy in this study, as shown by the PPV and NPV. The AdaBoost, XGBoost, and CART predicted more false-positive patients, resulting in lower accuracy and precision. The AUCs based on the testing set of the five ML algorithms are shown in Figure 2.

**Figure 3:**
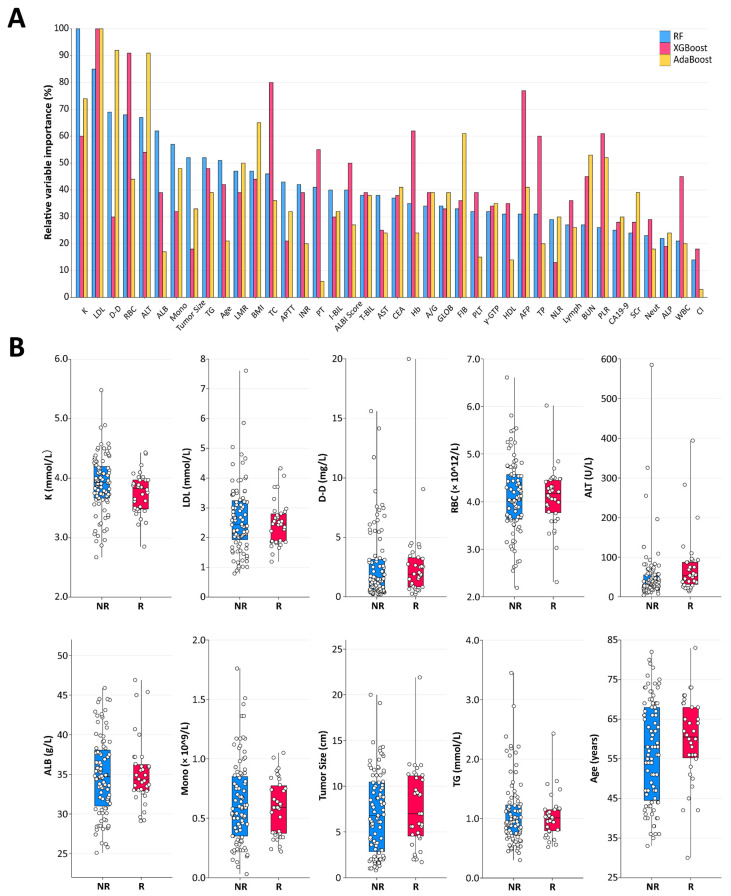
Importance matrix plot of the RF, AdaBoost, and XGBoost models. (**A**) Relative importance of the variables for segregation of the nonresponse group and response group calculated in the RF, AdaBoost, and XGBoost models. The variable importance is represented as a percentage of the highest value. (**B**) Box and jitter plots representing the distribution of the top 10 important features for distinguishing nonresponse and response. LDL, low-density lipoprotein; D-D, d-dimer; RBC, red blood cell; ALT, alanine aminotransferase; ALB, albumin; Mono, monocyte; TG, triglyceride; LMR, lymphocyte monocyte ratio; BMI, body mass index; TC, total cholesterol; APTT, activated partial thromboplastin time; INR, international normalized ratio; PT, prothrombin time; I-BIL, indirect bilirubin; ALBI, albumin–bilirubin; T-BIL, total bilirubin; AST, aspartate aminotransferase; CEA, car-cinoembryonic antigen; Hb, hemoglobin; A/G, albumin/globulin; GLOB, globulin; FIB, fibrinogen; PLT, platelet; γ-GTP, γ-glutamyl transpeptidase; HDL, high-density lipoprotein; AFP, alpha fetoprotein; TP, total protein; NLR, neutrophil lymphocyte ratio; Lymph, lymphocyte; BUN, blood urea nitrogen; PLR, platelet lymphocyte ratio; CA19-9, carbohydrate antigen 19-9; Scr, serum creatinine; Neut, neutrophil; ALP, alkaline phosphatase; WBC, white blood cell; NR, nonresponse; R, response.

The RF algorithm revealed the largest AUC (0.91), and the AUCs of the SVM, XGBoost, AdaBoost, and CART were 0.86, 0.80, 0.80, and 0.74, respectively. Then, we ranked the relative importance of the 40 features (Figure 3A) and identified the 10 most important predictors, including K, low-density lipoprotein (LDL), D-dimer (D-D), RBC, alanine aminotransferase (ALT), albumin (ALB), monocyte (Mono), tumor size, triglyceride (TG), and age. The box plots of the 10 predictor distributions between the nonresponse group and response group are shown in Figure 3B. The results before and after the unsupervised clustering of 125 patients with unresectable HCC are shown in the Supplementary File S1 (Figure S2).

### 3.3. Explainability of the Machine Learning Models

**Figure 4:**
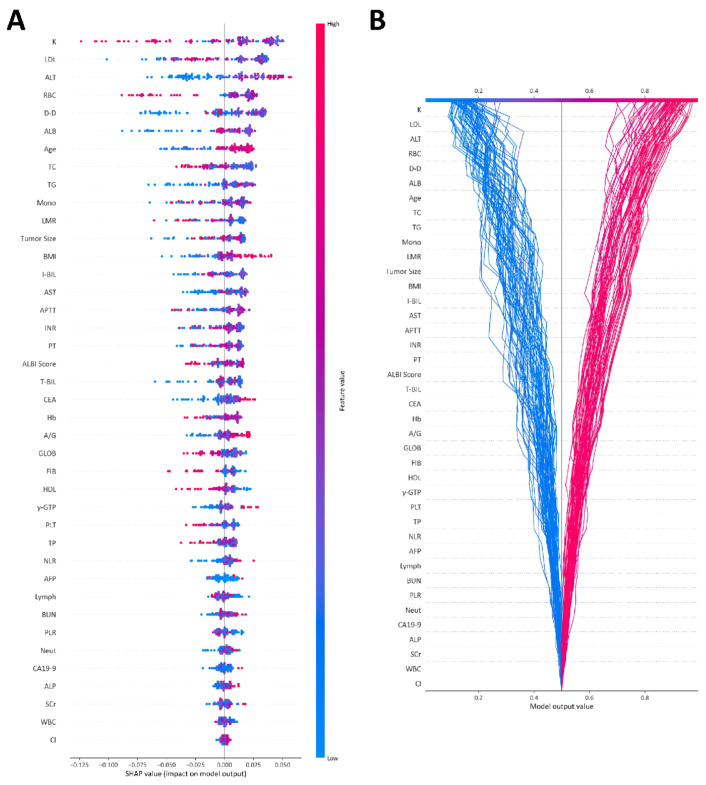

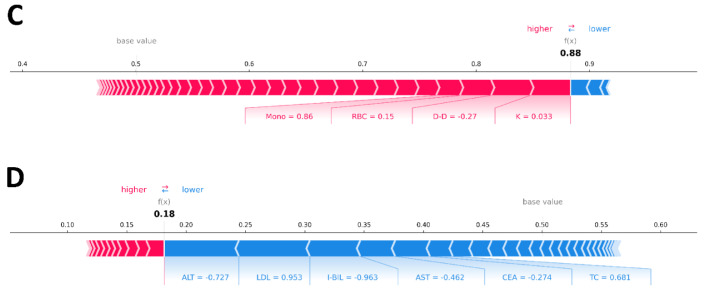
SHAP plot of the RF model: (**A**) SHAP summary plot of the top 40 features of the RF model; (**B**) SHAP decision plot of the top 40 features of the RF model; (**C**) SHAP force plot for the explanation of the model prediction results with a response sample from the testing set; (**D**) SHAP force plot for the explanation of the model prediction results with a nonresponse sample from the testing set. LDL, low-density lipoprotein; ALT, alanine aminotransferase; RBC, red blood cell; D-D, d-dimer; ALB, albumin; TC, total cholesterol; TG, triglyceride; Mono, monocyte; LMR, lymphocyte monocyte ratio; BMI, body mass index; I-BIL, indirect bilirubin; AST, aspartate aminotransferase; APTT, activated partial thromboplastin time; INR, international normalized ratio; PT, prothrombin time; ALBI, albumin–bilirubin; T-BIL, total bilirubin; CEA, carcinoembryonic antigen; Hb, hemoglobin; A/G, albumin/globulin; GLOB, globulin; FIB, fibrinogen; HDL, high-density lipoprotein; γ-GTP, γ-glutamyl transpeptidase; PLT, platelet; TP, total protein; NLR, neutrophil lymphocyte ratio; AFP, alpha fetoprotein; Lymph, lymphocyte; BUN, blood urea nitrogen; PLR, platelet lymphocyte ratio; Neut, neutrophil; CA19-9, carbohydrate antigen 19-9; ALP, alkaline phosphatase; Scr, serum creatinine; WBC, white blood cell; SHAP, Shapley Additive exPlanation.

To comprehensively explain the ML models, we used SHAP values to explain how the features affect the efficacy of lenvatinib combined with TACE. Since the SHAP algorithm could be applied to the tree-based algorithm for explanation but not for the SVM algorithm, we selected the RF model, which showed an excellent performance (AUC = 0.91), to calculate the SHAP values and draw a SHAP summary plot of the top 40 features (Figure 4A). Each given patient in the training set was represented by a single point for each feature on the plot. The x-axis coordinate of each point was determined by the SHAP value, and the points were stacked along each feature to show the density. The features were ordered by the mean absolute value of the SHAP values for each feature. The color was used to show the original value of a feature; red and blue indicated high and low probability values, respectively. For example, a lower serum K, higher ALT, older age, and larger tumor size and BMI suggested a higher probability of response to lenvatinib combined with TACE. The SHAP decision plot provided a detailed view of the inner workings of the RF model (Figure 4B). The gray, vertical line in the decision plot represents the base value of the model, while each colored line represented the prediction for each patient in the training set. Each prediction line showed how the SHAP values were accumulated from the base values at the bottom of the plot to the top of the plot to obtain the final score. The red line indicated a higher probability of response to lenvatinib combined with TACE, while the blue line indicated a lower probability. The SHAP decision plot provided a global explanation of the internal framework for the RF model. We then randomly selected one patient who had a response to lenvatinib combined with TACE (Figure 4C) and one patient who did not have a response (Figure 4D) in the testing set to illustrate the explainability of the model using a SHAP force plot. The arrows represented the effect of each feature on the predicted result, with the red arrows indicating an increased probability of response to the combined treatment and the blue arrows indicating a decreased probability. The predicted values were 0.88 for the first patient and 0.18 for the second patient after integrating the effects for each feature, which were in accordance with the true situation.

## 4. Discussion

HCC remains a serious challenge around the world, with a predicted case number of over one million by 2025, which is the third leading cause of cancer-related deaths [25]. ML has been shown to be effective in diagnosing and treating liver diseases for the purpose of precision therapy [26]. We performed a retrospective study based on the real world data from two hospitals. Then, we constructed a valuable clinical data-based model using ML methods to predict the response to lenvatinib combined with TACE for patients with unresectable HCC, which may provide new insights into risk prediction and clinical decision making.

Previous studies have shown that lenvatinib combined with TACE can significantly improve the OS and PFS compared with TACE monotherapy for patients with unresectable HCC [27,28]. Another study based on propensity score matching (PSM) confirmed the beneficial effect of alternating lenvatinib and TACE therapy compared with lenvatinib monotherapy on the prognosis of patients with intermediate stage HCC [29]. Similarly, a recent phase III randomized clinical trial in China demonstrated a longer OS and PFS and a higher ORR with lenvatinib combined with TACE therapy compared to lenvatinib monotherapy [30]. However, the chemotherapy resistance, complicated heterogeneity, and genetic aberrations of unresectable HCC result in a lower ORR and hinder the formulation of treatment options [31,32]. Identifying new clinical biomarkers is of great significance for clinical decision making and individual treatment.

Therefore, we conducted this retrospective study to explore the predictors of the response to lenvatinib combined with TACE using ML algorithms. We constructed five types of ML models, and all of the models showed excellent predictive performance, with AUCs of 0.74 to 0.91. The SVM and RF algorithms achieved the highest accuracy rate of 86.5%. The SVM and RF are both highly complicated models, and we could input data and obtain predictions but have no idea how they worked internally. Although the SVM showed the best predictive performance, explaining the model through hyperplanes is not conducive to clinical practice, because the kernel selected for performing the tuning is “rbf”, which is a nonlinear kernel, and the model could not provide the weights assigned to the features. Tree ensemble algorithms could provide the advantage of being more accurate and stable predictions compared to a single decision tree and the availability of ranking the importance of features. AdaBoost, XGBoost, and CART predicted more patients who did not have a response to lenvatinib combined with TACE as having a response in the testing set, resulting in an increase in false positives. The optimal performing tree ensemble algorithm in this study was the RF. However, it would be too complicated to apply the RF model to clinical practice directly. Since the SHAP algorithm could be applied to the tree-based algorithm for explanation but not to the SVM algorithm. We further calculated the SHAP values based on the RF model to present global and local explanations of the efficacy to understand the internal framework.

The association of serum K, age, BMI, and tumor size with the efficacy of lenvatinib combined with TACE for unresectable HCC has not been clearly evaluated in previous studies. In this study, the global explanation of the SHAP model demonstrated the trend that patients with lower serum K, older age, larger BMI, and larger tumor size were more likely to be responsive to combination therapy. Previous studies have demonstrated that ALB was associated with the efficacy and prognosis of lenvatinib treatment, which was considered a predictive marker [10,33]. Similarly, the platelet–lymphocyte ratio (PLR), neutrophil-to-lymphocyte ratio (NLR), and alpha fetoprotein (AFP) were also related to the survival of patients with unresectable HCC treated with lenvatinib, which were considered early predictors of objective response [34,35,36]. In our study, we obtained a similar result. In addition, we used the SHAP model to predict patient efficacy at the individualized level by force plot to support clinical decision making. A comparison of the output value and base value can be applied to determine whether patients tend to have a response to lenvatinib combined with TACE therapy.

To our knowledge, this is the first study to predict the efficacy of lenvatinib combined with TACE therapy for unresectable HCC using ML algorithms and SHAP methods. However, this study has some limitations. Firstly, the sample size included in the study was moderate, which is similar to previous studies [10,14]. However, ML algorithms potentially perform better on larger sample size datasets, as well as specializing in detecting a realistic relationship between features and outcomes. Secondly, there was a lack of an independent external validation cohort to test the accuracy and robustness of the models. Thirdly, some concealed relationships between features were likely to be neglected by the ML algorithms, and other neglected features that are considered clinically unimportant may be associated with the efficacy of lenvatinib combined with TACE. In addition, we need to collect multidimensional features for analysis to improve the predictive performance, including lifestyle habits, environmental factors, imaging markers, and pathological examination-related features.

## 5. Conclusions

A valuable model was developed using ML algorithms based on clinical features to predict the response to lenvatinib combined with TACE for patients with unresectable HCC. Combining ML with SHAP can provide a reasonable explanation of the efficacy prediction at the individualized level, which is important for clinical decision making.

## Figures and Tables

**Table 1:** Demographic characteristics, pretreatment serum biomarkers, and tumor characteristics of the training set and testing set.

	All Patients (*n* = 125)	Training Set (*n* = 88)	Testing Set (*n* = 37)	*p*-Value
**Demographic Characteristics**				
Age, median (IQR), y	58.0 (47.0, 68.0)	58.0 (46.3, 69.0)	59.0 (47.5, 67.5)	0.963
Gender				0.890
Male, *n* (%)	109 (87.2)	77 (87.5)	32 (86.5)	
Female, *n* (%)	16 (12.8)	11 (12.5)	5 (13.5)	
BMI, mean ± SD, kg/m^2^	22.0 ± 2.8	22.1 ± 2.6	21.8 ± 3.1	0.577
Smoking History				0.858
Yes, *n* (%)	42 (33.6)	30 (34.1)	12 (32.4)	
No, *n* (%)	83 (66.4)	58 (65.9)	25 (67.6)	
Drinking History				0.689
Yes, *n* (%)	44 (35.2)	30 (34.1)	14 (37.8)	
No, *n* (%)	81 (64.8)	58 (65.9)	23 (62.2)	
HBV Infection History				0.239
Yes, *n* (%)	78 (62.4)	52 (59.1)	26 (70.1)	
No, *n* (%)	47 (37.6)	36 (40.9)	11 (29.9)	
HCV Infection History				
Yes, *n* (%)	0 (0.0)	0 (0.0)	0 (0.0)	
No, *n* (%)	125 (100.0)	88 (100.0)	37 (100.0)	
Hypertensive History				0.683
Yes, *n* (%)	37 (29.6)	27 (30.7)	10 (27.0)	
No, *n* (%)	88 (70.4)	61 (69.3)	27 (73.0)	
Diabetes History				0.351
Yes, *n* (%)	18 (14.4)	11 (12.5)	7 (18.9)	
No, *n* (%)	107 (85.6)	77 (87.5)	30 (81.1)	
Heart Disease history				0.613
Yes, *n* (%)	2 (1.6)	2 (2.3)	0 (0.0)	
No, *n* (%)	123 (98.4)	86 (97.7)	37 (100.0)	
NAFLD History				
Yes, *n* (%)	0 (0.0)	0 (0.0)	0 (0.0)	
No, *n* (%)	125 (100.0)	88 (100.0)	37 (100.0)	
Cirrhosis, *n* (%)				0.623
Yes, *n* (%)	105 (84.0)	73 (83.0)	32 (86.5)	
No, *n* (%)	20 (16.0)	15 (17.0)	5 (13.5)	
ECOG-PS				0.547
Grade 0, *n* (%)	66 (52.8)	48 (54.5)	18 (48.6)	
Grade 1, *n* (%)	59 (47.2)	40 (45.5)	19 (51.4)	
**Pretreatment Serum Biomarkers**				
RBC, median (IQR), 10^12^/L	4.1 (3.7, 4.5)	4.2 (3.7, 4.5)	3.9 (3.5, 4.3)	0.048
WBC, median (IQR), 10^9^/L	6.3 (4.9, 8.0)	6.2 (5.1, 8.0)	6.5 (4.6, 7.9)	0.867
Neut, median (IQR), 10^9^/L	4.1 (2.9, 6.0)	4.2 (3.0, 5.9)	3.8 (2.4, 6.1)	0.400
Mono, median (IQR), 10^9^/L	0.6 (0.4, 0.8)	0.6 (0.3, 0.8)	0.6 (0.4, 0.9)	0.273
Lymph, median (IQR), 10^9^/L	1.1 (0.8,1.7)	1.1 (0.8, 1.6)	1.2 (0.8, 1.8)	0.253
Hb, mean ± SD, g/L	123.0 ± 22.7	124.3 ± 22.7	119.9 ± 22.4	0.322
PLT, median (IQR), 10^9^/L	167.0 (112.5, 252.0)	160.0 (109.3, 245.0)	180.0 (127.5, 271.5)	0.184
PLR, median (IQR)	138.3 (101.4, 219.3)	136.2 (96.4, 230.7)	140.4 (108.3, 214.2)	0.735
NLR, median (IQR)	3.7 (1.9, 5.9)	4.3 (2.0, 6.4)	3.0 (1.9, 4.2)	0.136
LMR, median (IQR)	2.0 (1.3, 3.6)	2.0 (1.3, 3.8)	1.8 (1.3, 3.5)	0.756
PT, median (IQR), s	14.5 (13.6, 15.5)	14.4 (13.5, 15.4)	14.7 (14.1, 16.1)	0.101
FIB, median (IQR), g/L	3.8 (2.9, 5.2)	3.8 (2.9, 5.2)	4.1 (3.0, 5.3)	0.766
APTT, median (IQR), s	39.3 (36.3, 44.0)	38.6 (36.2, 41.9)	41.7 (36.9, 48.5)	0.040
TT, median (IQR), s	17.3 (16.2, 18.4)	17.4 (16.2, 18.4)	16.9 (15.8, 18.5)	0.381
INR, median (IQR)	1.1 (1.0, 1.2)	1.1 (1.0, 1.2)	1.1 (1.1, 1.2)	0.101
D-D, median (IQR), mg/L	1.5 (0.7, 3.2)	1.3 (0.6, 3.2)	2.1 (0.8, 4.4)	0.072
AFP, median (IQR), ng/mL	161.4 (5.5, 3326.5)	113.2 (4.3, 2991.3)	277.7 (17.0, 3575.5)	0.318
CEA, median (IQR), μg/L	2.4 (1.6, 3.6)	2.4 (1.4,3.4)	2.6 (2.3, 4.1)	0.056
CA19-9, median (IQR), U/mL	19.9 (10.5, 41.9)	19.9 (9.7, 39.9)	19.9 (13.0, 32.0)	0.083
T-BIL, median (IQR), μmol/L	17.0 (11.5, 25.5)	15.5 (11.0,25.0)	19.0 (13.0, 32.0)	0.191
D-BIL, median (IQR), μmol/L	8.0 (5.5, 13.0)	7.0 (5.0, 12.0)	10.0 (6.0, 16.0)	0.029
I-BIL, median (IQR), μmol/L	8.0 (6.0, 12.0)	8.0 (6.0, 12.0)	8.0 (6.0, 12.5)	0.766
TP, mean ± SD, g/L	69.9 ± 8.4	69.4 ± 8.2	71.1 ± 8.6	0.300
ALB, median (IQR), g/L	34.6 (31.9, 37.6)	34.6 (32.1, 38.9)	34.7 (29.7, 36.3)	0.188
GLOB, median (IQR), g/L	34.1 (29.4, 38.7)	33.4 (29.4, 37.5)	36.8 (29.3, 42.8)	0.045
A/G, mean ± SD	1.0 ± 0.3	1.1 ± 0.3	1.0 ± 0.2	0.011
ALT, median (IQR), U/L	39.0 (25.0, 68.0)	38.5 (24.3, 63.5)	40.0 (27.0, 68.5)	0.918
AST, median (IQR), U/L	57.0 (39.0, 93.0)	56.0 (35.5, 90.5)	59.0 (41.5, 97.5)	0.496
ALP, median (IQR), U/L	182.0 (115.0, 233.5)	165.5 (112.5, 210.0)	210.0 (124.0, 272.0)	0.068
γ-GTP, median (IQR), U/L	182.0 (74.5, 257.5)	163.0 (61.3,247.5)	193.0 (103.5, 315.0)	0.232
BUN, median (IQR), mmol/L	5.1 (3.8, 6.4)	5.2 (3.8, 6.5)	5.0 (3.8, 5.8)	0.465
SCr, median (IQR), μmol/L	68.0 (56.5, 78.0)	67.0 (57.0, 80.3)	69.0 (55.0, 77.5)	0.762
K, mean ± SD, mmol/L	3.9 ± 0.5	3.8 ± 0.5	4.0 ± 0.4	0.154
Na, median (IQR), mmol/L	137.0 (135.0, 139.0)	137.0 (135.0, 139.0)	136.0 (133.5, 138.5)	0.052
Cl, median (IQR), mmol/L	102.0 (99.5, 105.0)	103.0 (100.0, 105.0)	101.0 (99.0, 104.0)	0.066
TC, median (IQR), mmol/L	4.3 (3.4, 5.0)	4.3 (3.5, 5.0)	4.2 (3.3, 5.0)	0.869
TG, median (IQR), mmol/L	1.0 (0.7, 1.2)	1.0 (0.7, 1.2)	0.9 (0.8, 1.4)	0.534
HDL, mean ± SD, mmol/L	0.9 ± 0.3	0.9 ± 0.3	0.8 ± 0.3	0.105
LDL, median (IQR), mmol/L	2.5 (1.9,3.2)	2.5 (1.8, 3.2)	2.4 (1.9, 3.2)	0.920
**Tumor Characteristics**				
ALBI Score, mean ± SD	−2.1 ± 0.5	−2.2 ± 0.5	−2.0 ± 0.4	0.053
Child–Pugh Score				0.493
A (5–6 scores), *n* (%)	80 (64.0)	58 (65.9)	22 (59.5)	
B (7 scores), *n* (%)	45 (36.0)	30 (34.1)	15 (40.5)	
T Stage				0.415
T1a, *n* (%)	4 (3.2)	4 (4.5)	0 (0.0)	
T1b, *n* (%)	10 (8.0)	7 (8.0)	3 (8.1)	
T2, *n* (%)	28 (22.4)	19 (21.6)	9 (24.3)	
T3, *n* (%)	40 (32.0)	31 (35.2)	9 (24.3)	
T4, *n* (%)	43 (34.4)	27 (30.7)	16 (43.3)	
N Stage				0.842
N0, *n* (%)	76 (60.8)	54 (61.4)	22 (59.5)	
N1, *n* (%)	49 (39.2)	34 (38.6)	15 (40.5)	
M Stage				0.351
M0, *n* (%)	107 (85.6)	77 (87.5)	30 (81.1)	
M1, *n* (%)	18 (14.4)	11 (12.5)	7 (18.9)	
TNM Stage				0.467
IA, *n* (%)	2 (1.6)	2 (2.3)	0 (0.0)	
IB, *n* (%)	9 (7.2)	7 (8.0)	2 (5.4)	
II, *n* (%)	17 (13.6)	9 (10.2)	8 (21.6)	
IIIA, *n* (%)	21 16.8)	17 (19.3)	4 (10.8)	
IIIB, *n* (%)	13 (10.4)	9 (10.2)	4 (10.8)	
IVA, *n* (%)	45 (36.0)	33 (37.5)	12 (32.4)	
IVB, *n* (%)	18 (14.4)	11 (12.5)	7 (18.9)	
BCLC Stage				0.584
A, *n* (%)	12 (9.6)	10 (11.4)	2 (5.4)	
B, *n* (%)	38 (30.4)	26 (29.5)	12 (32.4)	
C, *n* (%)	75 (60.0)	52 (59.1)	23 (62.2)	
Tumor Size, median (IQR), mm	6.9 (3.1, 11.1)	6.7 (2.9, 11.2)	7.0 (3.7, 9.9)	0.920
Tumor Number				0.361
Solitary, *n* (%)	23 (18.4)	18 (20.5)	5 (13.5)	
Multiple, *n* (%)	102 (81.6)	70 (79.5)	32 (86.5)	
Vascular Invasion, *n* (%)				0.177
Yes, *n* (%)	43 (34.4)	27 (30.7)	16 (43.2)	
No, *n* (%)	82 (65.6)	61 (69.3)	21 (56.8)	
Lymphatic Metastasis, *n* (%)				0.523
Yes, *n* (%)	52 (41.6)	35 (39.8)	17 (45.9)	
No, *n* (%)	73 (58.4)	53 (60.2)	20 (54.1)	
Distant Metastasis, *n* (%)				0.351
Yes, *n* (%)	18 (14.4)	11 (12.5)	7 (18.9)	
No, *n* (%)	107 (85.6)	77 (87.5)	30 (81.1)	

BMI, body mass index; HBV, hepatitis B virus; HCV, hepatitis C virus; NAFLD, nonalcohol fatty liver disease; ECOG-PS, Eastern Cooperative Oncology Group performance status; RBC, red blood cell; WBC, white blood cell; Neut, neutrophil; Mono, monocyte; Lymph, lymphocyte; Hb, hemoglobin; PLT, platelet; PLR, platelet lymphocyte ratio; NLR, neutrophil lymphocyte ratio; LMR, lymphocyte monocyte ratio; PT, prothrombin time; FIB, fibrinogen; APTT, activated partial thromboplastin time; TT, thrombin time; INR, international normalized ratio; D-D, d-dimer; AFP, alpha fetoprotein; CEA, carcinoembryonic antigen; CA19-9, carbohydrate antigen 19-9; T-BIL, total bilirubin; D-BIL, direct bilirubin; I-BIL, indirect bilirubin; TP, total protein; ALB, albumin; GLOB, globulin; A/G, albumin/globulin; ALT, alanine aminotransferase; AST, aspartate aminotransferase; ALP, alkaline phosphatase; γ-GTP, γ-glutamyl transpeptidase; BUN, blood urea nitrogen; Scr, serum creatinine; TC, total cholesterol; TG, triglyceride; HDL, high-density lipoprotein; LDL, low-density lipoprotein; ALBI, albumin–bilirubin; TNM, tumor node metastasis; BCLC, Barcelona Clinic Liver Cancer.

**Table 2:** Predictive performance matrix of five machine learning models.

Classifier	Accuracy	Precision	Recall	F1-Score	Sensitivity	Specificity	PPV	NPV	MCC	AUC (95% CI)
CART	78.4%	54.5%	66.7%	60.0%	66.7%	82.1%	54.5%	88.5%	0.46	0.74 (0.57–0.92)
Adaboost	78.4%	54.5%	66.7%	60.0%	66.7%	82.1%	54.5%	88.9%	0.46	0.80 (0.59–0.93)
XGBoost	81.1%	60.0%	66.7%	63.2%	66.7%	85.7%	60.0%	88.9%	0.51	0.80 (0.60–0.94)
SVM	86.5%	75.0%	66.7%	70.6%	66.7%	92.9%	75.0%	89.7%	0.62	0.86 (0.63–0.96)
RF	86.5%	75.0%	66.7%	70.6%	66.7%	92.9%	75.0%	89.7%	0.62	0.91 (0.61–0.95)

CART, classification and regression tree; AdaBoost, adaptive boosting; XGBoost, extreme gradient boosting; SVM, support vector machine; RF, random forest; PPV, positive predictive value; NPV, negative predictive value; MCC, Matthews correlation coefficient; AUC, area under curve; CI, confidence interval.

## Data Availability

All data and materials supporting the findings of this work are available from the corresponding author upon reasonable request.

## Supplementary Materials

The following supporting information can be downloaded at: https://www.mdpi.com/article/10.3390/cancers15030625/s1, File S1: The raw data of 125 patients with unresectable HCC; File S2: Figure S1: PCA plot of the two medical centers based on 64 standardized clinical features; Figure S2: The scatter plot before (A) and after (B) Kmeans clustering; Table S1: Demographic characteristics, pre-treatment serum biomarkers and tumor characteristics of non-response group and response group; Table S2: The best hyperparameters for five machine learning algorithms; Table S3: The confusion matrix for five machine learning algorithms.

## Abbreviations

HCC: hepatocellular carcinoma; PLC, primary liver cancer; TKI, tyrosine kinase inhibitor; ORR, objective response rate; OS, overall survival; PFS, progression-free survival; TACE, transarterial chemoembolization; VEGF, vascular endothelial growth factor; FGF, fibroblast growth factor; AI, artificial intelligence; ML, machine learning; APSAL, Asian Pacific Association for the Study of the Liver; ECOG-PS, Eastern Cooperative Oncology Group performance status; PCA, principal component analysis; mRECIST, modified RECIST; CR, complete response; PR, partial response; PD, progressive disease; SD, stable disease; DCR, disease control rate; BMI, body mass index; HBV, hepatitis B virus; HCV, hepatitis C virus; NAFLD, nonalcohol fatty liver disease; RF, random forest; RFE, recursive feature elimination; SMOTE, synthetic minority oversampling technique; CART, classification and regression tree; AdaBoost, adaptive boosting; XGBoost, extreme gradient boosting; SVM, support vector machine; ROC, receiver operating characteristic; AUC, area under curve; PPV, positive predictive value; NPV, negative predictive value; MCC, Matthews correlation coefficient; t-SNE, t-distributed stochastic neighbor embedding; SHAP, Shapley Additive exPlanation; SD, standard deviation; IQR, interquartile range; RBC, red blood cell; WBC, white blood cell; Neut, neutrophil; Mono, monocyte; Lymph, lymphocyte; Hb, hemoglobin; PLT, platelet; PLR, platelet–lymphocyte ratio; NLR, neutrophil–lymphocyte ratio; LMR, lymphocyte–monocyte ratio; PT, prothrombin time; FIB, fibrinogen; APTT, activated partial thromboplastin time; TT, thrombin time; INR, international normalized ratio; D-D, d-dimer; AFP, alpha fetoprotein; CEA, carcinoembryonic antigen; CA19-9, carbohydrate antigen 19-9; T-BIL, total bilirubin; D-BIL, direct bilirubin; I-BIL, indirect bilirubin; TP, total protein; ALB, albumin; GLOB, globulin; A/G, albumin/globulin; ALT, alanine aminotransferase; AST, aspartate aminotransferase; ALP, alkaline phosphatase; γ-GTP, γ-glutamyl transpeptidase; BUN, blood urea nitrogen; Scr, serum creatinine; TC, total cholesterol; TG, triglyceride; HDL, high-density lipoprotein; LDL, low-density lipoprotein; ALBI, albumin–bilirubin; TNM, tumor node metastasis; BCLC, Barcelona Clinic Liver Cancer; CI, confidence interval; PSM, propensity score matching.
