# Supplementary material for: Machine Learning to Predict the Response to Lenvatinib Combined with Transarterial Chemoembolization for Unresectable Hepatocellular Carcinoma
Source: Cancers (Basel). 2023 Jan 19;15(3):625. doi: 10.3390/cancers15030625 (PMC9913670; doi:10.3390/cancers15030625)
Supplement: Supplementary file 1 [file cancers-15-00625-s001.zip › Supplementary File S2.pdf]

# Supplementary File S2 Content


- **Supplementary File S2 Table S1-------------------------------------------------------------------------Page 2-7**
- **Supplementary File S2 Table S2---------------------------------------------------------------------------Page 8**
- **Supplementary File S2 Table S3---------------------------------------------------------------------------Page 9**
- **Supplementary File S2 Figure S1----------------------------------------------------------------------Page 10**
- **Supplementary File S2 Figure S2----------------------------------------------------------------------Page 11**

**Table S1.** Demographic characteristics, pre-treatment serum biomarkers and tumor characteristics of non-response group and response group.

| | **All patients (n=125)** | **Non-response Group (n = 91)** | **Response Group (n = 34)** | ***P* value** |
|---|---|---|---|---|
| **Demographic Characteristics** | | | | |
| Age, median (IQR), y | 58.0 (47.0, 68.0) | 58.0 (44.0, 68.0) | 60.5 (54.5, 68.3) | 0.285 |
| Gender | | | | 0.608 |
| Male, n (%) | 109 (87.2) | 78 (85.7) | 31 (91.2) | |
| Female, n (%) | 16 (12.8) | 13 (14.3) | 3 (8.8) | |
| BMI, mean ± SD, $kg/m^2$ | 22.0 ± 2.8 | 21.8 ± 2.7 | 22.4 ± 2.9 | 0.293 |
| Smoking History | | | | 0.502 |
| Yes, n (%) | 42 (33.6) | 29 (31.9) | 13 (38.2) | |
| No, n (%) | 83 (66.4) | 62 (68.1) | 21 (61.8) | |
| Drinking History | | | | 0.392 |
| Yes, n (%) | 44 (35.2) | 30 (33.0) | 14 (41.2) | |
| No, n (%) | 81 (64.8) | 61 (69.0) | 20 (58.8) | |
| HBV Infection History | | | | 0.745 |
| Yes, n (%) | 78 (62.4) | 56 (61.5) | 22 (64.7) | |
| No, n (%) | 47 (37.6) | 35 (38.5) | 12 (35.3) | |
| HCV Infection History | | | | / |
| Yes, n (%) | 0 (0.0) | 0 (0.0) | 0 (0.0) | |
| No, n (%) | 125 (100.0) | 91 (100.0) | 34 (100.0) | |
| Hypertensive History | | | | 0.074 |
| Yes, n (%) | 37 (29.6) | 31 (34.1) | 6 (17.6) | |
| No, n (%) | 88 (70.4) | 60 (65.9) | 28 (82.4) | |

| | | | | |
|---|---|---|---|---|
| Diabetes History | | | | 0.821 |
| Yes, n (%) | 18 (14.4) | 13 (14.3) | 5 (14.7) | |
| No, n (%) | 107 (85.6) | 78 (85.7) | 29 (85.3) | |
| Heart Disease | | | | 0.467 |
| Yes, n (%) | 2 (1.6) | 1 (1.1) | 1 (2.9) | |
| No, n (%) | 123 (98.4) | 90 (98.9) | 33 (97.1) | |
| NAFLD History | | | | / |
| Yes, n (%) | 0 (0.0) | 0 (0.0) | 0 (0.0) | |
| No, n (%) | 125 (100.0) | 91 (100.0) | 34 (0.0) | |
| Cirrhosis, n (%) | | | | 0.160 |
| Yes, n (%) | 105 (84.0) | 79 (86.8) | 26 (76.5) | |
| No, n (%) | 20 (16.0) | 12 (13.2) | 8 (23.5) | |
| ECOG-PS | | | | 0.701 |
| Grade 0, n (%) | 66 (52.8) | 49 (53.8) | 17 (50.0) | |
| Grade 1, n (%) | 59 (47.2) | 42 (46.2) | 17 (50.0) | |
| **Pre-treatment Serum Biomarkers** | | | | |
| RBC, median (IQR), 10^12/L | 4.1 (3.7, 4.5) | 4.1 (3.6, 4.6) | 4.2 (3.7, 4.5) | 0.954 |
| WBC, median (IQR), 10^9/L | 6.3 (4.9, 8.0) | 6.2 (4.9, 7.8) | 6.5 (4.8, 8.2) | 0.667 |
| Neut, median (IQR), 10^9/L | 4.1 (2.9, 6.0) | 4.0 (2.8, 5.8) | 4.3 (3.0, 6.3) | 0.348 |
| Mono, median (IQR), 10^9/L | 0.6 (0.4, 0.8) | 0.6 (0.4, 0.9) | 0.6 (0.4, 0.8) | 0.933 |
| Lymph, median (IQR), 10^9/L | 1.1 (0.8,1.7) | 1.1 (0.8, 1.8) | 1.0 (0.7, 1.5) | 0.363 |
| Hb, mean ± SD, g/L | 123.0 ± 22.7 | 122.5 ± 24.4 | 124.3 ± 17.5 | 0.663 |
| PLT, median (IQR), 10^9/L | 167.0 (112.5, 252.0) | 167.0 (112.0, 261.0) | 167.0 (114.0, 243.8) | 0.896 |

| | | | | |
|---|---|---|---|---|
| PLR, median (IQR) | 138.3 (101.4, 219.3) | 135.2 (105.6, 213.8) | 146.9 (88.1, 262.1) | 0.631 |
| NLR, median (IQR) | 3.7 (1.9, 5.9) | 3.3 (1.9, 5.6) | 4.5 (1.9, 6.7) | 0.171 |
| LMR, median (IQR) | 2.0 (1.3, 3.6) | 2.0 (1.3, 3.8) | 1.8 (1.2, 3.0) | 0.316 |
| PT, median (IQR), s | 14.5 (13.6, 15.5) | 14.7 (13.5, 15.7) | 14.4 (13.6, 15.2) | 0.394 |
| FIB, median (IQR), g/L | 3.8 (2.9, 5.2) | 3.8 (2.8, 5.4) | 3.8 (3.0, 5.1) | 0.907 |
| APTT, median (IQR), s | 39.3 (36.3, 44.0) | 39.1 (36.2, 44.7) | 39.5 (36.3, 42.7) | 0.788 |
| TT, median (IQR), s | 17.3 (16.2, 18.4) | 17.3 (16.2, 18.5) | 17.4 (15.9, 18.1) | 0.566 |
| INR, median (IQR) | 1.1 (1.0, 1.2) | 1.1 (1.0, 1.2) | 1.1 (1.0, 1.2) | 0.394 |
| D-D, median (IQR), mg/L | 1.5 (0.7, 3.2) | 1.3 (0.6, 3.2) | 1.9 (0.8, 3.4) | 0.340 |
| AFP, median (IQR), ng/mL | 161.4 (5.5, 3326.5) | 205.3 (8.2, 4149.0) | 26.9 (3.7, 1680.9) | 0.212 |
| CEA, median (IQR), μg/L | 2.4 (1.6, 3.6) | 2.4 (1.5, 3.5) | 2.4 (1.9, 4.1) | 0.598 |
| CA19-9, median (IQR), U/mL | 19.9 (10.5, 41.9) | 19.9 (10.7, 42.8) | 19.9 (8.9, 42.9) | 0.430 |
| T-BIL, median (IQR), μmol/L | 17.0 (11.5, 25.5) | 17.0 (11.0, 26.0) | 15.5 (12.0, 25.3) | 0.912 |
| D-BIL, median (IQR), μmol/L | 8.0 (5.5, 13.0) | 8.0 (5.0, 13.0) | 7.0 (5.8, 14.3) | 0.996 |
| I-BIL, median (IQR), μmol/L | 8.0 (6.0, 12.0) | 8.0 (6.0, 13.0) | 8.0 (6.0, 11.0) | 0.753 |
| TP, mean ± SD, g/L | 69.9 ± 8.4 | 70.6 ± 8.5 | 68.2 ± 7.6 | 0.152 |
| ALB, median (IQR), g/L | 34.6 (31.9, 37.6) | 34.7 (30.7, 38.3) | 34.3 (32.9, 36.5) | 0.824 |
| GLOB, median (IQR), g/L | 34.1 (29.4, 38.7) | 35.4 (29.4, 39.1) | 32.4 (29.6, 36.1) | 0.148 |
| A/G, mean ± SD | 1.0 ± 0.3 | 1.0 ± 0.3 | 1.1 ± 0.2 | 0.151 |
| ALT, median (IQR), U/L | 39.0 (25.0, 68.0) | 35.0 (24.0, 56.0) | 54.5 (29.8, 88.5) | 0.010 |
| AST, median (IQR), U/L | 57.0 (39.0, 93.0) | 54.0 (37.0, 83.0) | 68.0 (45.0, 113.5) | 0.080 |
| ALP, median (IQR), U/L | 182.0 (115.0, 233.5) | 180.0 (116.0, 244.0) | 200.0 (109.5, 217.8) | 0.866 |
| γ-GTP, median (IQR), U/L | 182.0 (74.5, 257.5) | 159.0 (64.0, 252.0) | 203.0 (82.0, 313.3) | 0.314 |

| | | | | |
|---|---|---|---|---|
| BUN, median (IQR), mmol/L | 5.1 (3.8, 6.4) | 5.3 (3.8, 6.1) | 4.7 (3.9, 7.7) | 0.951 |
| SCr, median (IQR), μmol/L | 68.0 (56.5, 78.0) | 69.0 (57.0, 78.0) | 62.0 (56.0, 76.5) | 0.379 |
| K, mean ± SD, mmol/L | 3.9 ± 0.5 | 3.9 ± 0.5 | 3.7 ± 0.3 | 0.044 |
| Na, median (IQR), mmol/L | 137.0 (135.0, 139.0) | 137.0 (134.0, 139.0) | 137.0 (135.0,139.3) | 0.503 |
| Cl, median (IQR), mmol/L | 102.0 (99.5, 105.0) | 102.0 (99.0, 105.0) | 102.5 (100.0, 105.3) | 0.513 |
| TC, median (IQR), mmol/L | 4.3 (3.4, 5.0) | 4.4 (3.4, 5.3) | 4.1 (3.4, 4.7) | 0.297 |
| TG, median (IQR), mmol/L | 1.0 (0.7, 1.2) | 0.9 (0.7, 1.2) | 1.0 (0.8, 1.2) | 0.651 |
| HDL, mean ± SD, mmol/L | 0.9 ± 0.3 | 0.9 ± 0.3 | 0.9 ± 0.3 | 0.877 |
| LDL, median (IQR), mmol/L | 2.5 (1.9,3.2) | 2.5 (1.8, 3.3) | 2.5 (1.9, 2.8) | 0.598 |
| **Tumor Characteristics** | | | | |
| ALBI Score, mean ± SD | -2.1 ± 0.5 | -2.1 ± 0.5 | -2.2 ± 0.5 | 0.849 |
| Child-Pugh Score | | | | 0.750 |
| A (5-6 scores), n (%) | 80 (64.0) | 59 (64.8) | 21 (61.8) | |
| B (7 scores), n (%) | 45 (36.0) | 32 (35.2) | 13 (38.2) | |
| T Stage | | | | 0.404 |
| T1a, n (%) | 4 (3.2) | 2 (2.2) | 2 (5.9) | |
| T1b, n (%) | 10 (8.0) | 6 (6.6) | 4 (11.8) | |
| T2, n (%) | 28 (22.4) | 23 (25.3) | 5 (14.7) | |
| T3, n (%) | 40 (32.0) | 27 (29.6) | 13 (38.2) | |
| T4, n (%) | 43 (34.4) | 33 (36.3) | 10 (29.4) | |
| N Stage | | | | 0.585 |
| N0, n (%) | 76 (60.8) | 54 (59.3) | 22 (64.7) | |
| N1, n (%) | 49 (39.2) | 37 (40.7) | 12 (35.3) | |

| | | | | |
|---|---|---|---|---|
| M Stage | | | | 0.358 |
| M0, n (%) | 107 (85.6) | 80 (87.9) | 27 (79.4) | |
| M1, n (%) | 18 (14.4) | 11 (12.1) | 7 (20.6) | |
| TNM Stage | | | | 0.448 |
| IA, n (%) | 2 (1.6) | 1 (1.1) | 1 (2.9) | |
| IB, n (%) | 9 (7.2) | 5 (5.5) | 4 11.8) | |
| II, n (%) | 17 (13.6) | 14 (15.4) | 3 (8.8) | |
| IIIA, n (%) | 21 16.8) | 14 (15.4) | 7 (20.6) | |
| IIIB, n (%) | 13 (10.4) | 11 (12.1) | 2 (5.9) | |
| IVA, n (%) | 45 (36.0) | 35 (38.5) | 10 (29.4) | |
| IVB, n (%) | 18 (14.4) | 11 (12.1) | 7 (20.6) | |
| BCLC Stage | | | | 0.172 |
| A, n (%) | 12 (9.6) | 6 (6.6) | 6 (17.6) | |
| B, n (%) | 38 (30.4) | 29 (31.9) | 9 (26.5) | |
| C, n (%) | 75 (60.0) | 56 (61.5) | 19 (55.9) | |
| Tumor Size, median (IQR), mm | 6.9 (3.1, 11.1) | 6.8 (2.7, 10.5) | 7.0 (4.5, 11.2) | 0.478 |
| Tumor Number | | | | 0.894 |
| Solitary, n (%) | 23 (18.4) | 17 (18.7) | 6 (17.6) | |
| Multiple, n (%) | 102 (81.6) | 74 (81.3) | 28 (82.4) | |
| Vascular Invasion, n (%) | | | | 0.473 |
| Yes, n (%) | 43 (34.4) | 33 (36.3) | 10 (29.4) | |
| No, n (%) | 82 (65.6) | 58 (63.7) | 24 (70.6) | |
| Lymphatic Metastasis, n (%) | | | | 0.641 |

| | | | | |
|---|---|---|---|---|
| Yes, n (%) | 52 (41.6) | 39 (42.9) | 13 (38.2) | |
| No, n (%) | 73 (58.4) | 52 (57.1) | 21 (61.8) | |
| Distant Metastasis, n (%) | | | | 0.358 |
| Yes, n (%) | 18 (14.4) | 11 (12.1) | 7 (20.6) | |
| No, n (%) | 107 (85.6) | 80 (87.9) | 27 (79.4) | |

Abbreviation: BMI, body mass index; HBV, hepatitis B virus; HCV, hepatitis C virus; NAFLD, non-alcohol fatty liver disease; ECOG-PS, Eastern Cooperative Oncology Group performance status; RBC, red blood cell; WBC, white blood cell; Neut, neutrophil; Mono, monocyte; Lymph, lymphocyte; Hb, hemoglobin; PLT, platelets; PLR, platelet lymphocyte ratio; NLR, neutrophil lymphocyte ratio; LMR, lymphocyte monocyte ratio; PT, prothrombin time; FIB, fibrinogen; APTT, activar.ted partial thromboplastin time; TT, thrombin time; INR, international normalized ratio; D-D, d-dimer; AFP, Alpha feto-protein; CEA, carcinoembryonic antigen; CA19-9, carbohydrate antigen 19-9; T-BIL, total bilirubin; D-BIL, direct bilirubin; I-BIL, indirect bilirubin; TP, total protein; ALB, albumin; GLOB, globulin; A/G, albumin/globulin; ALT, alanine aminotransferase; AST, aspartate aminotransferase; ALP, alkaline phosphatase; γ-GTP, γ-glutamyl transpeptidase; BUN, blood urea nitrogen; Scr, serum creatinine; TC, total cholesterol; TG, triglyceride; HDL, high-density lipoprotein; LDL, low-density lipoprotein; ALBI, albumin-bilirubin; TNM, Tumor Node Metastasis; BCLC, Barcelona Clinic Liver Cancer.

**Table S2.** The best hyperparameters for five machine learning algorithms.

| Machine Learning Algorithms | Best Hyperparameters |
|---|---|
| CART | criterion= 'gini'<br>max_depth=9<br>min_samples_split=3<br>random_state=420 |
| AdaBoost | n_estimators=800<br>random_state=420<br>learning_rate=1.0 |
| XGBoost | min_child_weight=1<br>max_depth=15<br>learning_rate=0.1<br>gamma=0.0<br>colsample_bytree=0.3<br>random_state=420 |
| SVM | gamma=0.2<br>C=10<br>kernel='rbf' |
| RF | n_estimators=500<br>random_state=420<br>criterion='gini'<br>max_depth=10 |

| | |
|---|---|
| | max_features='auto' |

Abbreviation: CART, classification and regression tree; AdaBoost, adaptive boosting; XGBoost, extreme gradient boosting; RF, random forest; SVM, support vector machine.

**Table S3.** The confusion matrix for five machine learning algorithms.

| **Machine Learning Algorithm** | **Predicted Labels** | **Real Labels** | |
|---|---|---|---|
| | | Response | Non-response |
| CART | Response | 6 | 5 |
| | Non-response | 3 | 23 |
| AdaBoost | Response | 6 | 5 |
| | Non-response | 3 | 23 |
| XGBoost | Response | 6 | 4 |
| | Non-response | 3 | 24 |
| RF | Response | 6 | 2 |
| | Non-response | 3 | 26 |
| SVM | Response | 6 | 2 |
| | Non-response | 3 | 26 |

Abbreviation: CART, classification and regression tree; AdaBoost, adaptive boosting; XGBoost, extreme gradient boosting; RF, random forest; SVM, support vector machine.

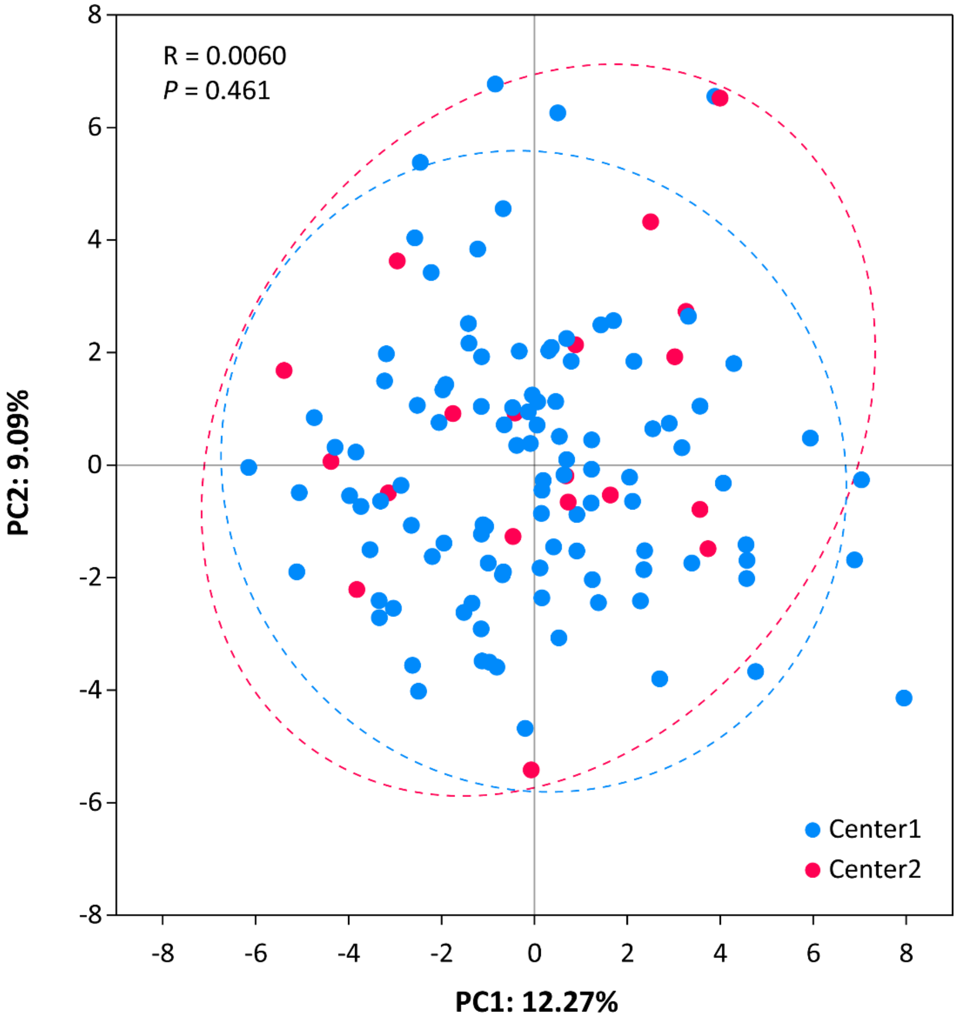

**Figure S1.** PCA plot of the two medical centers based on 64 standardized clinical features.

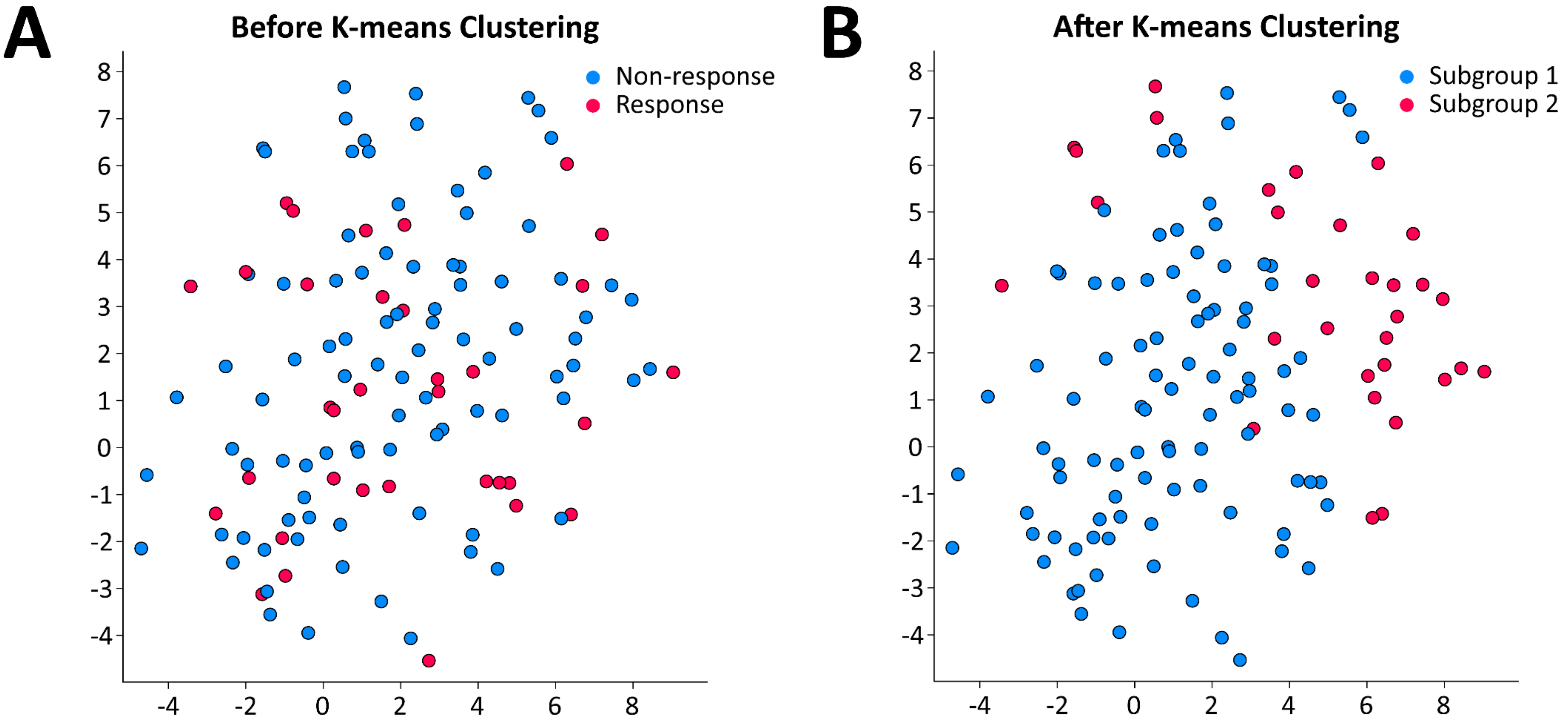


**Figure S2.** The scatter plot before (A) and after (B) Kmeans clustering.
